# Effects of PPIs use on clinical outcomes of urothelial cancer patients receiving immune checkpoint inhibitor therapy

**DOI:** 10.3389/fphar.2022.1018411

**Published:** 2022-09-26

**Authors:** Lilong Zhang, Chen Chen, Dongqi Chai, Chunlei Li, Tianrui Kuang, Li Liu, Keshuai Dong, Wenhong Deng, Weixing Wang

**Affiliations:** Department of General Surgery, Renmin Hospital of Wuhan University, Wuhan, China

**Keywords:** immune checkpoint inhibitors, proton pump inhibitors, urothelial cancer, clinical outcomes, meta-analysis

## Abstract

**Objective:** Immune checkpoint inhibitors (ICIs) have recently demonstrated promising performance in improving the prognosis of urological cancer patients. The goal of this meta-analysis was to determine the impact of PPI use on the clinical outcomes of urological cancer patients receiving ICI therapy.

**Methods:** Before 6 May 2022, the eligible literature was searched using PubMed, EMBASE, Cochrane Library, and Google Scholar. The clinical outcomes were overall survival (OS), progression-free survival (PFS), and objective response rate (ORR).

**Results:** A total of six articles met the inclusion criteria, and of the 1980 patients with advanced or metastatic urothelial cancers (UC) included. The meta-analysis displayed that PPI use could increase the risk of progression by 50.7% (HR: 1.507, 95% CI: 1.327–1.711, *p* < 0.001) and death by 58.7% (HR: 1.587, 95% CI: 1.367–1.842, *p* < 0.001), and reduce the ORR (OR: 0.503, 95% CI: 0.360–0.703, *p* < 0.001) in UC patients receiving ICIs. No significant heterogeneity and publication bias existed. Sensitivity analysis proved that the results were stable and reliable.

**Conclusion:** The meta-analysis indicated that concomitant PPI use was significantly associated with low clinical benefit in UC patients.

## 1 Introduction

Urological cancers, mostly including renal cell carcinoma (RCC), prostate cancer (PC), and urothelial cancer (UC), are the common public health concerns worldwide (Sung et al., 2021). Despite the advances in treatments and techniques for tumors, such as chemotherapy and molecular targeted therapy, the clinical prognosis of urological cancers has not improved considerably over the last 2 decades (Niu et al., 2021). The introduction of immune checkpoint inhibitors (ICIs) has transformed the treatment of a variety of cancers, including urological malignancies. These antibodies act by blocking the checkpoint pathways, which are physiologic mechanisms established to switch off the immune response and prevent autoimmunity (Bimbatti et al., 2022).

UC has the fourth highest rate of mutations of all cancers and is known to be highly antigenic (Kim et al., 2020), whereas RCC has a moderate tumor mutation load but a high frequency of deletion and clonal insertion mutations, which may be linked to neoantigen abundance and CD8^+^ T cell activation (Carretero-González et al., 2020). These characteristics make the theme appropriate for ICI therapy. In contrast, PC immunogenicity is hampered by a low mutation burden and a highly immunosuppressive microenvironment. As a result, it is deemed a “cold tumor” that is difficult to treat with ICIs (Kim and Koo, 2020). ICIs have been approved for RCC and UC and have been shown to improve patient survival when compared to traditional treatments (Pierantoni et al., 2019; Xu et al., 2020). However, the clinical efficacy of ICIs varies widely amongst sufferers, with only a tiny percentage of the population benefiting from treatment. Furthermore, primary resistance to ICIs is still frequent, and a significant number of patients continue to worsen or relapse as a result of ICI resistance (Sharma et al., 2017; Seto et al., 2019). Regrettably, no perfect biomarker for predicting the efficacy of ICIs exists at this time. Thus, the search for prospective biomarkers that predict its efficacy as well as factors that influence its efficacy is critical for a more targeted selection of treatment populations in clinical practice.

Antacid agents such as proton pump inhibitors (PPIs) and histamine-2-receptor antagonists (H2RAs) are commonly prescribed for extended periods in urological cancer patients. Recent evidence also suggested that PPI usage in patients with advanced NSCLC receiving ICI therapy was associated with an increased mortality risk (Qin et al., 2021; Rizzo et al., 2022; Wei et al., 2022). However, the relationship between antacid use and ICI outcomes in urological cancer patients remains controversial due to a lack of comprehensive evaluations. Therefore, we conducted the first systematic review and meta-analysis to elucidate whether antacid use affects the efficacy of ICI therapy for urological cancer. This will provide evidence for future clinical use of antacids in urological cancers treated with ICIs, thereby maximizing the clinical benefit to patients.

## 2 Materials and methods

### 2.1 Literature search strategies

This meta-analysis accompanied the Preferred Reporting Items for Systematic Reviews and Meta-Analyses (PRISMA) guidelines (Page et al., 2021). The protocol for this meta-analysis is available in PROSPERO (CRD42022332633). On 6 May 2022, PubMed (https://pubmed.ncbi.nlm.nih.gov/), EMBASE (https://www.embase.com/), and Cochrane Library (https://www.cochranelibrary.com/) were retrieved. The following Medical Subject Headings (MeSH) terms and their entry terms: “Immune Checkpoint Inhibitors” [Mesh], “Antacids” [Mesh], “Proton Pump Inhibitors” [Mesh], “Histamine H2 Antagonists” [Mesh], as well as the following terms: “omeprazole,” “pantoprazole,” “lansoprazole,” “esomeprazole,” “dexlansoprazole,” “rabeprazole,” “ranitidine” were searched in [All Fields]. Detailed search strategies were shown in Supplementary Table S1. We also searched Google Scholar to uncover gray literature that was not indexed in the previously listed databases, such as presentations and unpublished research data. Furthermore, we also manually retrieved the reference lists of eligible papers.

### 2.2 Study selection criteria

If articles matched all the following criteria, they were included (Sung et al., 2021). patients diagnosed with urological cancers (Niu et al., 2021); patients treated with ICIs (Bimbatti et al., 2022); patients separated into the antacid use group and non-antacid use group (Kim et al., 2020); provided at least one of the outcomes of interest [multivariable/adjusted overall survival (OS), progression-free survival (PFS), and objective response rate (ORR)]. For retrospective studies, the results of univariable analysis are vulnerable to confounding factors, so we included studies that provided multivariable analysis. Only the article with the most comprehensive data and rigorous methods was chosen when studies reported overlapping patient populations. Meanwhile, the following exclusion criteria were employed: abstract, comments, and case report.

### 2.3 Data extraction and quality assessment

Data extraction mainly focused on the author, publication year, study region, study period, study type, cancer type, the number of patients, the age of patients, the number of male patients, timing of antacid use, types of ICI treatment, types of antacids, and the outcomes of interest (OS, PFS, and ORR). Response Evaluation Criteria in Solid Tumors (RECIST) version 1.1 was used to estimate the ORR. The Newcastle-Ottawa Scale (NOS) score was used to estimate the quality of the retrospective studies (Wells et al., 2019). Literature with a score ≥7 was considered to be of high quality. Two authors independently cross-checked all the above steps, and the senior authors (Wenhong Deng and Wang Weixing) addressed any disparities.

### 2.4 Statistical methods

Stata MP16.0 was used for the statistical analysis. The HR and its 95% CI were used to calculate the influence of antacid use on the risk of survival in cancer patients. The association between ICI efficacy and antacid usage was expressed as an odds ratio (OR) with a 95% CI. The statistical heterogeneity among the studies was determined using the chi-squared test. *p* > 0.1 and I^2^ < 50% indicated low heterogeneity where a fixed-effect model was used; otherwise, the random-effect model was adopted. To reduce the influence of heterogeneity on the meta-analysis, a subgroup analysis was performed. Begg’s and Egger’s tests were implemented to assess publication bias. Sensitivity analysis by the leave-one-out method was conducted to estimate the stability of the results. All *p* values were two-sided with significance set at *p* < 0.05.

## 3 Results

### 3.1 Studies retrieved and characteristics

**FIGURE 1:**
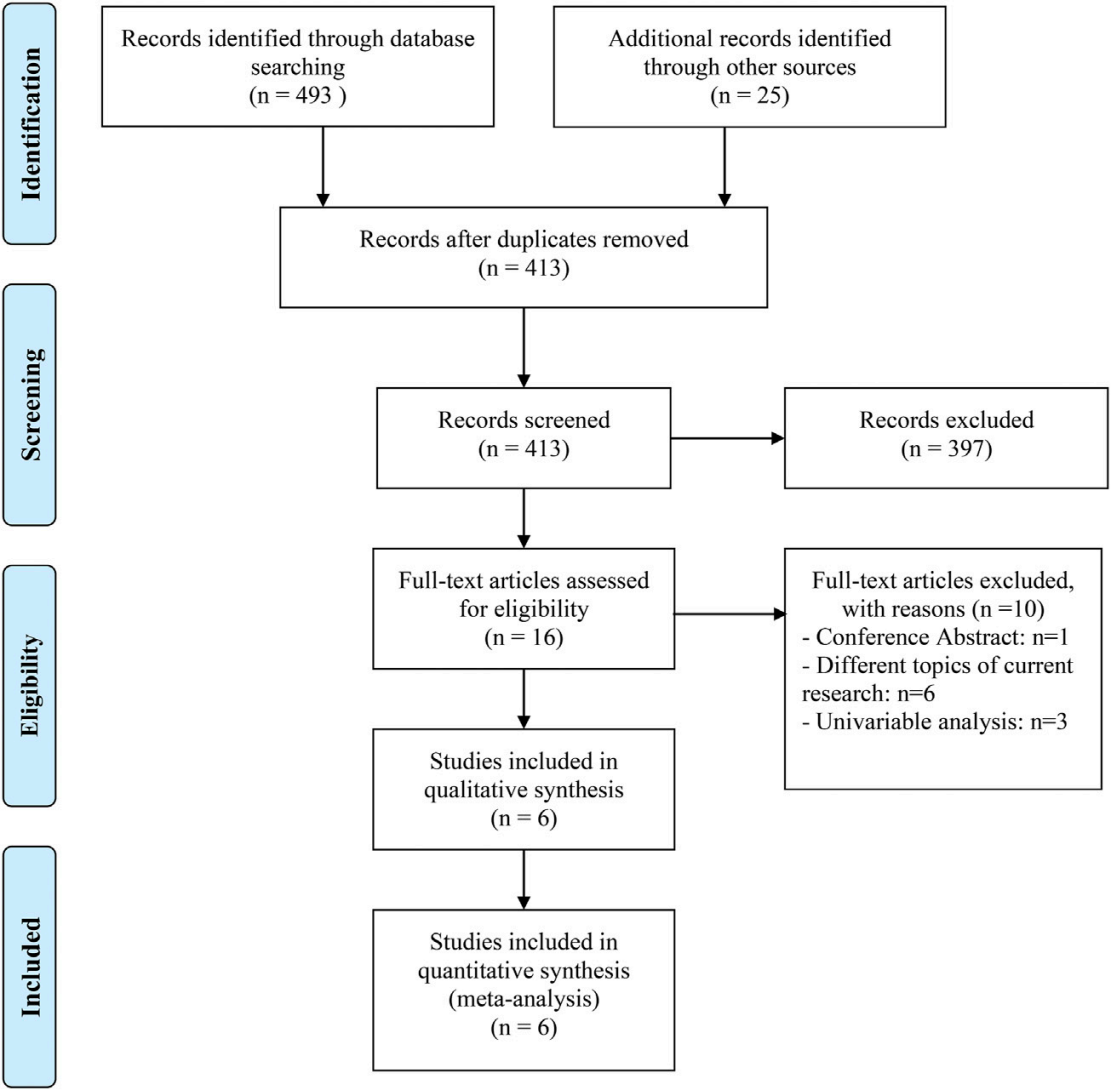
The flow diagram of identifying eligible studies.

We gathered 518 potentially eligible records and assessed their titles and abstracts to see if they were suitable for inclusion. We discovered that six articles (Hopkins et al., 2020; Ruiz-Bañobre et al., 2021; Fukuokaya et al., 2022; Kunimitsu et al., 2022; Okuyama et al., 2022; Tomisaki et al., 2022) met our criteria for inclusion after carefully reading the full texts of 16 records. The studies on RCC by Peng et al. (2022), Mollica et al. (2022), Kostine et al. (2021) only provided the results of univariate analysis, so they were excluded. Figure 1 depicts the flow diagram for identifying eligible studies. All six articles explored the effects of PPIs on ICI efficacy in patients with advanced or metastatic UC. A total of 1980 patients were included. Of the six retrospective studies, five articles were awarded seven or eight points and were regarded as high quality; one article was awarded six points and was deemed as medium quality. Table 1 shows the baseline characteristics of the included studies as well as the quality evaluation.

**TABLE 1:** Baseline characteristics of included studies.

Author, year	Study region	Study period	Study type	Cancer type	Antacids/Non-antacids			Timing of antacid use	Types of ICI treatment	Types of antacids	Quality
					No. of patients	Man	Age^a^				
Tomisaki, (2022)	Japan	03/2018–03/2021	R	Advanced UC	15/25	10/20	72/72	Within 60 days before and after beginning ICIs	Pembrolizumab	PPI	7
Ruiz-Bañobre, (2021), Peng et al. (2022)	Europe	06/2016–02/2020	R	Advanced or metastatic UC	54/65	45/51	70/68	Within 30 days before beginning ICIs	Pembrolizumab, Nivolumab, Atezolizumab, Durvalumab	PPI	7
Okuyama, (2022), Kunimitsu et al. (2022)	Japan	08/2015–04/2021	R	Advanced UC	99/56	75/43	71/73	Within 30 days before ICI initiation and during ICI therapy	Pembrolizumab, Nivolumab, Atezolizumab, Durvalumab	PPI	6
Kunimitsu et al. (2022), Ruiz-Bañobre et al. (2021)	Japan	05/2017–12/2020	R	Metastatic or Unresectable UC	34/45	24/35	72/71	Within 60 days before and 30 days after beginning ICIs	Pembrolizumab	PPI	7
Hopkins et al. (2020), Kostine et al. (2021)	Worldwide	—	R	Advanced or metastatic UC	471/889	359/696	67/67	Within 30 days before and after beginning ICIs	Atezolizumab	PPI	8
Fukuokaya et al. (2022), Hopkins et al. (2020)	Japan	04/2018–04/2021	R	Metastatic UC	86/141	62/103	70/71	Within 30 days before and after beginning ICIs	Pembrolizumab	PPI	7

a median/mean age; UC, urothelial carcinoma; R, retrospective study; PPI, proton pump inhibitor; ICI, immune checkpoint inhibitor; CTLA-4, the cytotoxic T-lymphocyte antigen-4; PD-(L)-1, programmed cell death protein (ligand)-1

### 3.2 Progression-free survival

**FIGURE 2:**
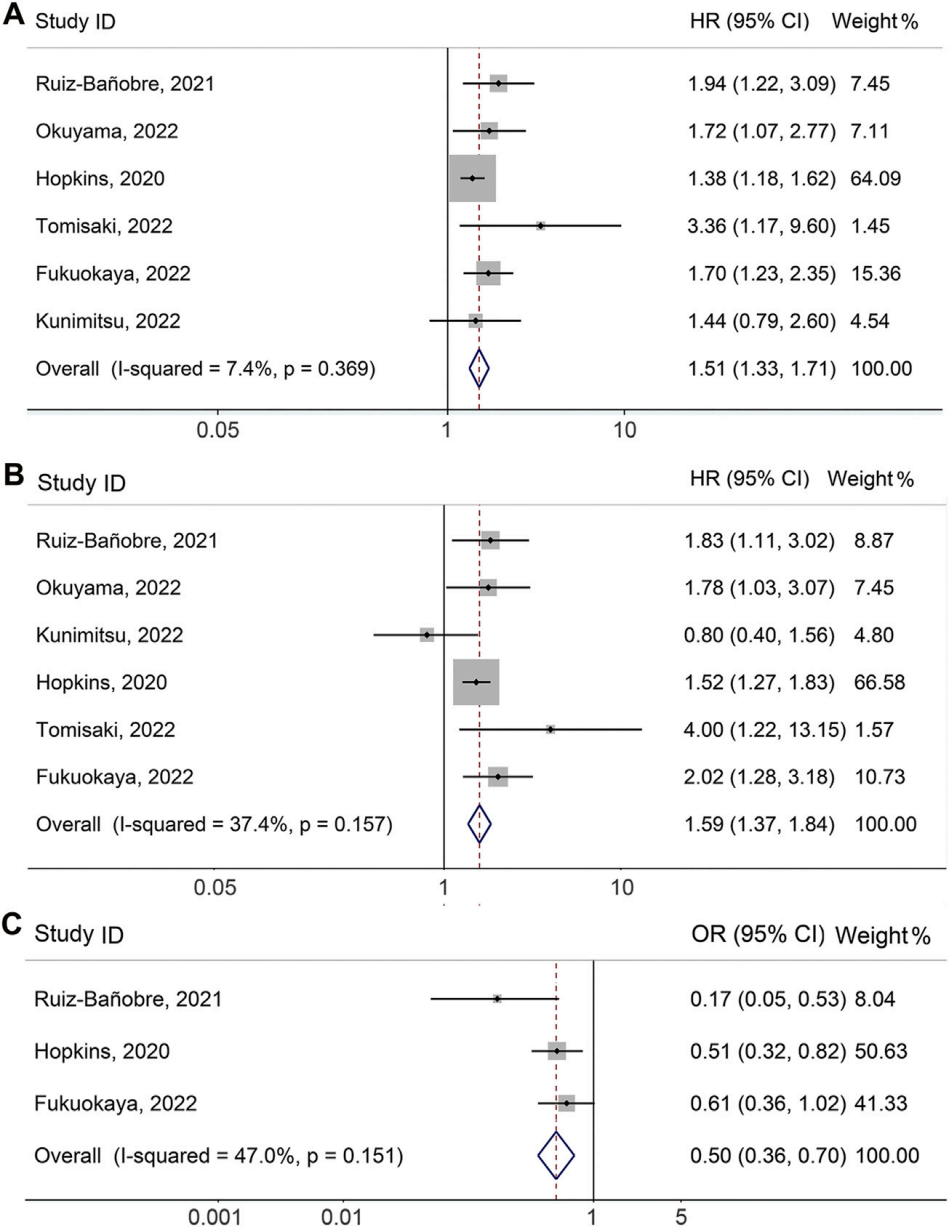
Forest plots of HR for correlation of proton pump inhibitor use with adjusted progression-free survival **(A)** and overall survival **(B)**. Forest plots of OR for correlation of proton pump inhibitor use with adjusted objective response rate **(C)**. OR, odds ratio; HR, hazard ratio; CL, confidence interval.

Six studies (Hopkins et al., 2020; Ruiz-Bañobre et al., 2021; Fukuokaya et al., 2022; Kunimitsu et al., 2022; Okuyama et al., 2022; Tomisaki et al., 2022), involving 1980 participants (759 who received PPIs and 1221 who did not), explored the impact of concomitant PPI usage on adjusted PFS among UC cancers receiving ICI treatment. As shown in Figure 2A, there was no significant heterogeneity among studies (I^2^ = 7.4%, *p* = 0.369), so a fixed-effects model was utilized. Compared with patients without PPI usage, the meta-analysis showed that PPI use could increase the risk of progression by 50.7% (Figure 2A, HR: 1.507, 95% CI: 1.327-1.711, *p* < 0.001).

### 3.3 Overall survival

\The meta-analysis of adjusted OS was performed on six studies (Hopkins et al., 2020; Ruiz-Bañobre et al., 2021; Fukuokaya et al., 2022; Kunimitsu et al., 2022; Okuyama et al., 2022; Tomisaki et al., 2022) with a total of 1980 participants (759 with PPIs and 1221 without PPIs). Since there was no significant heterogeneity (Figure 2B, I^2^ = 37.4%, *p* = 0.157), we applied a fixed-effects model. The meta-analysis revealed that PPI use was related to a shorter OS of UC patients receiving ICIs. PPI usage increased the risk of death by 58.7% (Figure 2B, HR: 1.587, 95% CI: 1.367–1.842, *p* < 0.001).

### 3.4 Objective response rate

As shown in Figure 2C, the pooled meta-analysis for multivariable analysis of the ORR included three studies (Hopkins et al., 2020; Ruiz-Bañobre et al., 2021; Fukuokaya et al., 2022) with 1706 urological cancer patients (611 with PPIs and 1095 without PPIs). No significant heterogeneity existed, so a fixed-effects model was implemented (I^2^ = 47.0%, *p* = 0.151). The results were consistent with the above finding that concomitant PPI use was associated with lower ORR in patients (OR: 0.503, 95% CI: 0.360–0.703, *p* < 0.001).

### 3.5 Publication bias

The Begg’s and Egger’s tests were then performed to investigate publication bias, with the results indicating that there was no evidence of publication bias for adjusted OS (Egger’s test: *p* = 0.574, Begg’s test: *p* = 1.000) and adjusted ORR (Egger’s test: *p* = 0.247, Begg’s test: *p* = 1.000) across the studies. However, Egger’s test showed a publication bias in adjusted PFS (Egger’s test: *p* = 0.032, Begg’s test: *p* = 0.452). Next, the number of missing studies in adjusted PFS was calculated using the trim and fill method. The combined HR was recalculated by including those missing hypothesis studies, which were not found to be significantly altered (HR:1.437, 95% CI: 1.277–1.617; *p* < 0.001). Thus, the publication bias had little effect, and the result was relatively stable.

### 3.6 Sensitivity analysis

**FIGURE 3:**
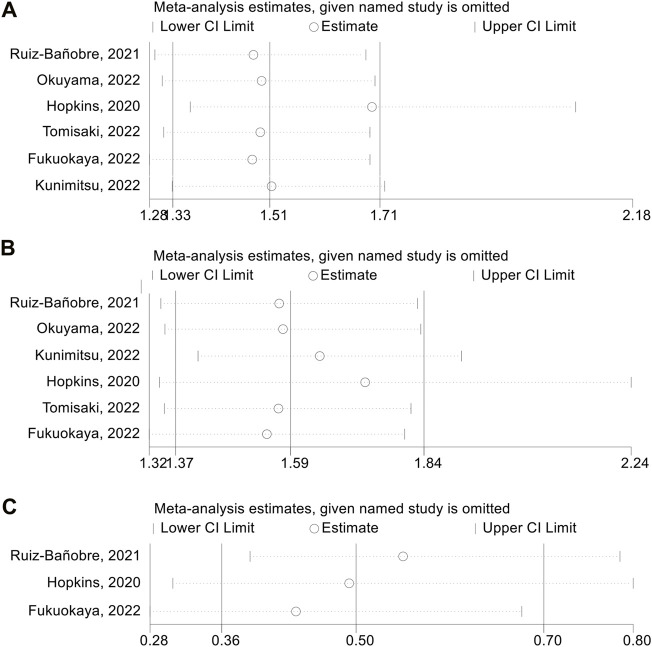
Sensitivity analysis of adjusted progression-free survival **(A)**, overall survival **(B)** and objective response rate **(C)**. CL, confidence interval.

We also performed a sensitivity analysis via the leave-one-out method to assess the impact of each study on the overall meta-analysis. As shown in Figure 3A, the pooled HR for adjusted PFS was not significantly changed after excluding one study at a time, ranging from 1.489 (95% CI: 1.311–1.692, after omitting Tomisaki, 2022) to 1.697 (95% CI: 1.359–2.076, after omitting Hopkins, 2020). Besides, the pooled HR for adjusted OS also did not significantly differ in the sensitivity analysis. The overall HR ranged from 1.542 (95% CI: 1.317–1.805, after omitting Fukuokaya et al., 2022) to 1.730 (95% CI: 1.336–2.238, after omitting Hopkins et al., 2020) (Figure 3B). Similarly, the pooled OR for adjusted ORR was not significantly different in the sensitivity analysis. The overall OR ranged from 0.439 (95% CI: 0.283–0.679, after omitting Fukuokaya et al., 2022) to 0.553 (95% CI: 0.390–0.784, after omitting Ruiz-Bañobre et al., 2021) (Figure 3C).

## 4 Discussion

With the increased use of ICIs in urological tumor therapy, tremendous effort has been made to uncover possible factors that affect its efficacy. Whether PPIs can impact the response to ICIs in UC patients is still being debated. For all we know, this is the first meta-analysis to investigate the relationship between PPIs and ICI efficacy in patients with UC. We synthesized all the available evidence and found concomitant PPI use was significantly associated with low clinical benefit in UC patients treated with ICIs. Our publication bias and sensitivity analyses verified the dependability of our conclusions. Consequently, our study is essential and hopes to provide novel insights into the precise management of PPIs in clinical practice. PPIs should be used with caution before and after ICI treatment in patients with UC.

PPIs were not only used to treat gastrointestinal adverse effects (nausea and vomiting) caused by systemic antineoplastic therapy; they were also used prophylactically for cancer patients taking high-dose glucocorticoids as an antiemetic regimen and with concomitant non-steroidal anti-inflammatory drugs as analgesics. Besides, tumor patients with a history of peptic ulcers or bleeding used PPI prophylaxis to reduce the incidence of stress ulcers (Triadafilopoulos et al., 2013). PPIs have been demonstrated to impact the intestinal microbiota, owing to both altered stomach acidity and direct compounds effects (Imhann et al., 2016; Le Bastard et al., 2018; Maier et al., 2018; Reveles et al., 2018). A significant decrease in bacterial richness and specific bacteria, such as the *Bifidobacteriaceae* and *Ruminococcaceae*, as well as a remarkable increase in pathogenic bacteria, were found among PPI users compared to non-users in a study of 1,815 people (Imhann et al., 2016; Reveles et al., 2018). Currently, the impact of microbiota on the response to ICI treatment is receiving increasing attention. Two landmark studies in mice provided the first evidence that the microbiome had a direct impact on ICI effectiveness (Sivan et al., 2015; Vétizou et al., 2015). Prospective studies have also revealed that microbiome diversity and composition were strongly associated with the efficacy of ICIs in patients with RCC (Derosa et al., 2020; Salgia et al., 2020) and NSCLC (Huemer et al., 2019; Hakozaki et al., 2020), among others. Dysbiosis of the gut microbiota reduces the activity of ICIs (Sivan et al., 2015; Vétizou et al., 2015; Huemer et al., 2019; Derosa et al., 2020; Hakozaki et al., 2020; Salgia et al., 2020). Furthermore, several preclinical studies have revealed that PPIs could impair the physiological function of natural killer cells, cytotoxic T-lymphocytes, and polymorphonuclear neutrophils, all of which are implicated in the efficacy of ICIs (Aybay et al., 1995; Zedtwitz-Liebenstein et al., 2002). Thus, PPIs may reduce the efficacy of ICIs by altering the intestinal flora and affecting innate immune cell function.

However, there is also evidence that PPIs not only inhibit tumor growth and enhance chemosensitivity by modulating the acidic environment, but also promote immune responses and prevent tumor immune escape (Peppicelli et al., 2015; Spugnini and Fais, 2017). Esomeprazole has also been shown to inhibit melanoma growth by inactivating NF-κB to downregulate vascular endothelial growth factor-C (VEGF-C) expression (Peppicelli et al., 2013). Notably, no basic research has been conducted on the role of PPI in the development of UC. In the context of ICI treatment, the underlying mechanisms of the effects of PPI on UC are completely unknown and need to be investigated in subsequent experiments.

This article has some inherent restrictions, to be sure. To begin with, this study was essentially a meta-analysis that relied on previously published articles. We did not have sufficient data to perform subgroup analyses based on different types, and doses of PPIs and ICIs, the PPI window respective to ICIs start, etc. Secondly, all included articles in this meta-analysis are retrospective studies with intrinsic limitations of reporting and selection bias. Thus, a larger prospective study should be performed to better understand the relationship between PPI use and ICI efficacy.

## 5 Conclusion

The meta-analysis suggested that concomitant PPI use was significantly associated with low clinical benefit in UC patients.

## Data Availability

The original contributions presented in the study are included in the article/Supplementary Material, further inquiries can be directed to the corresponding authors.
