# Supplementary material for: Effects of PPIs use on clinical outcomes of urothelial cancer patients receiving immune checkpoint inhibitor therapy
Source: Front Pharmacol. 2022 Sep 26;13:1018411. doi: 10.3389/fphar.2022.1018411 (PMC9549125; doi:10.3389/fphar.2022.1018411)
Supplement: Supplementary file 1 [file DataSheet1.docx]

1. **Detailed search strategies in Pubmed**

**(((((((((((((((((Antacids) OR (Alkalinizing Agents)) OR (Agents, Alkalinizing)) OR (Antacid)) OR (Alkalinizing Agent)) OR (Agent, Alkalinizing)) OR ("Antacids"[Mesh])) OR (((((((((Proton Pump Inhibitors) OR (Inhibitors, Proton Pump)) OR (Proton Pump Inhibitor)) OR (Inhibitor, Proton Pump)) OR (Pump Inhibitor, Proton)) OR (PPI)) OR (PPIs)) OR ("Proton Pump Inhibitors"[Mesh])) OR (("Histamine H2 Antagonists"[Mesh]) OR (((((((((((((((((((((((((((Histamine H2 Antagonists) OR (H2 Antagonists, Histamine)) OR (H2 Receptor Blockaders)) OR (Histamine H2 Antagonist)) OR (Blockaders, H2 Receptor)) OR (Antagonist, Histamine H2)) OR (H2 Antagonist, Histamine)) OR (H2 Receptor Blockader)) OR (Blockader, H2 Receptor)) OR (Receptor Blockader, H2)) OR (Receptor Blockaders, H2)) OR (Blockaders, Histamine H2 Receptor)) OR (Histamine H2 Receptor Blockader)) OR (Antihistaminics, H2)) OR (H2 Antihistaminics)) OR (Receptor Antagonists, Histamine H2)) OR (Histamine H2 Receptor Antagonists)) OR (Antagonists, Histamine H2)) OR (Histamine H2 Blockers)) OR (Blockers, Histamine H2)) OR (H2 Blockers, Histamine)) OR (Histamine H2 Blocker)) OR (Blocker, Histamine H2)) OR (H2 Blocker, Histamine)) OR (Histamine H2 Receptor Blockaders)) OR (Histamine H2 Receptor Antagonist)))))) OR (omeprazole)) OR (pantoprazole)) OR (lansoprazole)) OR (rabeprazole)) OR (esomeprazole)) OR (dexlansoprazole)) OR (rabeprazole)) OR (histamine-2-receptor antagonists)) OR (ranitidine)) AND (((((((((((((((((((((((((((((((((((((Immune Checkpoint Inhibitors) OR (Checkpoint Inhibitors, Immune)) OR (Immune Checkpoint Inhibitor)) OR (Checkpoint Inhibitor, Immune)) OR (Immune Checkpoint Blockers)) OR (Checkpoint Blockers, Immune)) OR (Immune Checkpoint Blockade)) OR (Checkpoint Blockade, Immune)) OR (Immune Checkpoint Inhibition)) OR (Checkpoint Inhibition, Immune)) OR (PD-L1 Inhibitors)) OR (PD L1 Inhibitors)) OR (PD-L1 Inhibitor)) OR (PD L1 Inhibitor)) OR (Programmed Death-Ligand 1 Inhibitors)) OR (Programmed Death Ligand 1 Inhibitors)) OR (PD-1-PD-L1 Blockade)) OR (Blockade, PD-1-PD-L1)) OR (PD 1 PD L1 Blockade)) OR (CTLA-4 Inhibitors)) OR (CTLA 4 Inhibitors)) OR (CTLA-4 Inhibitor)) OR (CTLA 4 Inhibitor)) OR (Cytotoxic T-Lymphocyte-Associated Protein 4 Inhibitors)) OR (Cytotoxic T Lymphocyte Associated Protein 4 Inhibitors)) OR (Cytotoxic T-Lymphocyte-Associated Protein 4 Inhibitor)) OR (Cytotoxic T Lymphocyte Associated Protein 4 Inhibitor)) OR (PD-1 Inhibitors)) OR (PD 1 Inhibitors)) OR (PD-1 Inhibitor)) OR (Inhibitor, PD-1)) OR (PD 1 Inhibitor)) OR (Programmed Cell Death Protein 1 Inhibitor)) OR (Programmed Cell Death Protein 1 Inhibitors)) OR (ICI)) OR (ICIs)) OR ("Immune Checkpoint Inhibitors"[Mesh]))**

1. **Detailed search strategies in Cochrane Library**

#1 MeSH descriptor: [Antacids] explode all trees

#2 (Antacids) OR (Alkalinizing Agents) OR (Agents, Alkalinizing) OR (Antacid) OR (Alkalinizing Agent)

#3 (Agent, Alkalinizing)

#4 MeSH descriptor: [Proton Pump Inhibitors] explode all trees

#5 (Inhibitors, Proton Pump) OR (Proton Pump Inhibitors) OR (Proton Pump Inhibitor) OR (Inhibitor, Proton Pump) OR (Pump Inhibitor, Proton)

#6 (PPI) OR (PPIs)

#7 MeSH descriptor: [Histamine H2 Antagonists] explode all trees

#8 (Histamine H2 Antagonists) OR (H2 Antagonists, Histamine) OR (H2 Receptor Blockaders) OR (Histamine H2 Antagonist) OR (Blockaders, H2 Receptor)

#9 (Antagonist, Histamine H2) OR (H2 Antagonist, Histamine) OR (H2 Receptor Blockader) OR (Blockader, H2 Receptor) OR (Receptor Blockader, H2)

#10 (Receptor Blockaders, H2) OR (Blockaders, Histamine H2 Receptor) OR (Histamine H2 Receptor Blockader) OR (Antihistaminics, H2) OR (H2 Antihistaminics)

#11 (Receptor Antagonists, Histamine H2) OR (Histamine H2 Receptor Antagonists) OR (Antagonists, Histamine H2) OR (Histamine H2 Blockers) OR (Blockers, Histamine H2)

#12 (H2 Blockers, Histamine) OR (Histamine H2 Blocker) OR (Blocker, Histamine H2) OR (H2 Blocker, Histamine) OR (Histamine H2 Receptor Blockaders)

#13 (Histamine H2 Receptor Antagonist) OR (omeprazole) OR (pantoprazole) OR (lansoprazole) OR (rabeprazole)

#14 (esomeprazole) OR (dexlansoprazole) OR (rabeprazole) OR (ranitidine)

#15 #1 OR #2 OR #3 OR #4 OR #5 OR #6 OR #7 OR #8 OR #9 OR #10 OR #11 OR #12 OR #13 OR #14

#16 MeSH descriptor: [Immune Checkpoint Inhibitors] explode all trees

#17 (Immune Checkpoint Inhibitors) OR (Checkpoint Inhibitors, Immune) OR (Immune Checkpoint Inhibitor) OR (Checkpoint Inhibitor, Immune) OR (Immune Checkpoint Blockers)

#18 (Immune Checkpoint Inhibitors) OR (Immune Checkpoint Blockade) OR (Checkpoint Blockade, Immune) OR (Immune Checkpoint Inhibition) OR (Checkpoint Inhibition, Immune)

#19 (PD-L1 Inhibitors) OR (PD L1 Inhibitors) OR (PD-L1 Inhibitor) OR (PD L1 Inhibitor) OR (Programmed Death-Ligand 1 Inhibitors)

#20 (Programmed Death Ligand 1 Inhibitors) OR (PD 1 PD L1 Blockade) OR (CTLA-4 Inhibitors) OR (CTLA 4 Inhibitors) OR (CTLA-4 Inhibitor)

#21 (CTLA 4 Inhibitor) OR (Cytotoxic T-Lymphocyte-Associated Protein 4 Inhibitors) OR (Cytotoxic T Lymphocyte Associated Protein 4 Inhibitors) OR (Cytotoxic T-Lymphocyte-Associated Protein 4 Inhibitor) OR (Cytotoxic T Lymphocyte Associated Protein 4 Inhibitor)

#22 (Programmed Cell Death Protein 1 Inhibitors) OR (ICI) OR (ICIs)

#23 #16 OR #17 OR #18 OR #19 OR #20 OR #21 OR #22

#24 #15 AND #23

1. **Detailed search strategies in EMBASE**

#1 'antacid agent'/exp

#2 'proton pump inhibitor'/exp

#3 'histamine h2 receptor antagonist'/exp

#4 antacid OR 'antacid drug' OR 'antacidic acid' OR 'antacidic agent' OR antacids OR 'antigastralgic agent' OR 'proton pump inhibitors' OR 'hydrogen potassium adenosine triphosphatase inhibitor' OR 'hydrogen potassium atpase inhibitor' OR 'antihistamines, h2' OR 'h 2 receptor blocking agent' OR 'h2 antagonist' OR 'h2 blocker' OR 'h2 blocking agent' OR 'h2 receptor antagonist' OR 'h2 receptor blocker' OR 'h2 receptor blocking agent' OR 'histamine 2 receptor antagonist' OR 'histamine 2 receptor blocker' OR 'histamine 2 receptor blocking agent' OR 'histamine h 2 receptor antagonist' OR 'histamine h2 antagonist' OR 'histamine h2 antagonists' OR 'histamine h2 blocker' OR 'histamine h2 blocking agent' OR 'histamine h2 receptor blockaders' OR 'histamine h2 receptor blocker' OR 'histamine h2 receptor blocking agent'

#5 'immune checkpoint inhibitor'/exp

#6 'immune checkpoint inhibitors' OR 'immune checkpoint inhibitor' OR (checkpoint AND inhibitor, AND immune) OR (immune AND checkpoint AND blockers) OR (checkpoint AND blockers, AND immune) OR (immune AND checkpoint AND blockade) OR (checkpoint AND blockade, AND immune) OR (immune AND checkpoint AND inhibition) OR (checkpoint AND inhibition, AND immune) OR ('pd l1' AND inhibitors) OR (pd AND l1 AND inhibitors) OR ('pd l1' AND inhibitor) OR (pd AND l1 AND inhibitor) OR (programmed AND 'death ligand' AND 1 AND inhibitors) OR (programmed AND death AND ligand AND 1 AND inhibitors) OR (1 AND pd AND l1 AND blockade) OR ('ctla 4' AND inhibitors) OR ('ctla 4' AND inhibitor) OR (ctla AND 4 AND inhibitors) OR (ctla AND 4 AND inhibitor) OR (cytotoxic AND t AND lymphocyte AND associated AND protein AND 4 AND inhibitors) OR (cytotoxic AND 't lymphocyte associated' AND protein AND 4 AND inhibitors) OR (cytotoxic AND 't lymphocyte associated' AND protein AND 4 AND inhibitor) OR (cytotoxic AND t AND lymphocyte AND associated AND protein AND 4 AND inhibitor) OR ('pd 1' AND inhibitors) OR ('pd 1' AND inhibitor) OR (inhibitor, AND 'pd 1') OR (pd AND 1 AND inhibitor) OR (programmed AND cell AND death AND protein AND 1 AND inhibitor) OR (programmed AND cell AND death AND protein AND 1 AND inhibitors) OR (immune AND checkpoint AND inhibitors)

#7 #1 OR #2 OR #3 OR #4

#8 #5 OR #6

#9 #7 OR #8
